# Real-world outcomes of venetoclax and azacitidine in Japanese patients with newly diagnosed acute myeloid leukemia (VENUS study)

**DOI:** 10.1007/s12185-025-04093-y

**Published:** 2025-11-07

**Authors:** Ryota Imanaka, Hiroki Numata, Yuna Katsuoka, Nobuhiko Uoshima, Satoru Hara, Jun Ando, Shuichi Ota, Goichi Yoshimoto, Akihito Matsuoka, Tetsuo Morita, Atsuko Tsutsui, Mizuha Kosugi-Kanaya, Tatsunori Goto

**Affiliations:** 1 Department of Hematology, Hiroshima Red Cross and Atomic-Bomb Survivors Hospital, Hiroshima, Japan; 2 Department of Hematology, Ebina General Hospital, Ebina, Japan; 3 Department of Hematology, National Hospital Organization Sendai Medical Center, Sendai, Japan; 4 Department of Hematology, Japanese Red Cross Kyoto Daini Hospital, Kyoto, Japan; 5 Department of Hematology, Chiba Rosai Hospital, Ichihara, Japan; 6 Department of Hematology, Juntendo University School of Medicine, Tokyo, Japan; 7 Department of Hematology, Sapporo Hokuyu Hospital, Sapporo, Japan; 8 Department of Hematology, Saga-Ken Medical Centre Koseikan, Saga, Japan; 9 Department of Hematology, Sakaide City Hospital, Sakaide, Japan; 10 AbbVie GK, Tokyo, Japan; 11 Department of Hematology, Japanese Red Cross Aichi Medical Center Nagoya Daiichi Hospital, 3-35 Michishita-cho, Nakamura-ku, Nagoya, 453-8511 Japan

**Keywords:** Venetoclax, Azacitidine, Acute myeloid leukemia, Myelodysplasia

## Abstract

Venetoclax (VEN) with azacitidine (AZA) is the standard treatment for patients with acute myeloid leukemia (AML) who are ineligible for intensive chemotherapy. However, real-world evidence on dosing, scheduling, and outcomes is lacking, particularly for patients with prior myelodysplastic syndrome (MDS) or AZA treatment, who have been excluded from clinical trials. This was a multicenter retrospective study of VEN + AZA in 120 patients newly diagnosed with AML between June 2021 and September 2022. The cohort had a median age of 77 years, 52% had secondary AML, 74% had previously been diagnosed with MDS, and 39% had previously received AZA. During cycle 1, half of the patients received 400 mg of VEN for a median of 27 days, with a median holding period of 12 days. With a median follow-up of 13.6 months, the rate of complete remission (CR) or CR with incomplete blood count recovery was 56.7% in VEN + AZA-treated patients in the overall cohort and 56.5% in patients with prior MDS. Median overall survival was 14.8 months for the overall cohort and 15.4 months for those with prior MDS. The real-world outcomes were comparable to those of clinical trials.

**Supplementary Information:**

The online version contains supplementary material available at 10.1007/s12185-025-04093-y.

## Introduction

Acute myeloid leukemia (AML) is a clonal hematopoietic malignancy that is characterized by high levels of molecular and pathogenic heterogeneity [1]. The prevalence of AML increases with age, and the median age at diagnosis is 68 years. The estimated 5-year survival differs significantly with age: it is < 19% for patients who are > 60 years of age and ~ 50% for younger patients [2, 3]. The outcomes remain suboptimal in older patients, most of whom are not candidates for standard intensive chemotherapy (IC).

The B-cell lymphoma 2 (BCL-2) inhibitor venetoclax (VEN) was approved for use in combination with azacitidine (AZA) in Japan in March 2021 for patients with newly diagnosed AML who are aged ≥ 75 years and/or are ineligible for IC [4]. In the VIALE-A study, VEN + AZA therapy improved the median overall survival (OS) from 9.6 to 14.7 months compared to AZA + placebo. Since this study was published, VEN + AZA has become the standard treatment for patients with AML who are unsuitable for IC [4–8]. However, there is a lack of evidence regarding the outcomes of patients treated with VEN + AZA in routine clinical practice including patients who were not included in clinical trials. For instance, myelodysplastic syndrome (MDS)-related AML is a common subtype in older patients [9, 10], but patients with a history of treatment for myeloid malignancy, including MDS, were excluded from the VIALE-A trial [4].

The classification related to MDS-related AML, which includes antecedent MDS, has been modified by newly incorporating myelodysplastic-related gene mutation patterns. Most of them are reclassified into AML myelodysplasia-related (AML-MR) in the International Consensus Classification, which is now included into genetically defined AML [9–12]. However, until commercial gene testing was implemented from March 2025, recently few facilities in Japan were able to test for myelodysplasia-related genes. Therefore in practice, the WHO 2016 AML with myelodysplasia-related changes (AML-MRC) classification is still widely used in clinical practice. In practice, the use of a category of “AML-MRC” [13], which includes patients with a history of MDS or MDS/myeloproliferative neoplasms, AML with multi-lineage dysplasia, and *de novo* AML with MDS-related cytogenetic abnormalities [14, 15], may still be warranted.

Patients with AML and a history of MDS, or AML-MRC, are frequently encountered in routine clinical practice and are associated with a poor prognosis. Furthermore, these patients often present severe cytopenia due to the underlying background of myelodysplasia [16]. Further evidence is needed on effectiveness and treatment management in patients with AML-MRC treated with VEN + AZA.

In addition, neutropenia is a common adverse event associated with VEN, and it is typically managed by protocol-recommended VEN dose modifications [17, 18]. We have recently demonstrated that the neutrophil counts of such patients who recover after a complete remission (CR) is achieved, through recommended dose schedule modifications and use of supportive care, including the administration of granulocyte colony-stimulating factor (G-CSF) [19]. Neutropenia management with VEN + AZA, including dose interruptions, the role of G-CSF use, and the concomitant use of CYP3A4 inhibitors particularly in patients with MDS-related AML, who are prone to severe cytopenia, are important issues that remain to be fully elucidated.

Although previous studies have demonstrated the clinical outcomes of VEN therapy in patients with AML in real-world settings [20–23], most available evidence does not focus on patients with MDS-related AML. Therefore, in the present study, we aimed to describe the treatment received, the use of supportive care, the remission rate, the survival, and the clinical factors associated with outcomes in patients with AML and a history of MDS.

## Materials and methods

### Patients

The VENUS study was a multicenter, retrospective, observational, chart review study of patients with newly diagnosed AML who were ineligible for IC and began VEN treatment between June 23, 2021 and September 30, 2022 at 10 sites in Japan. Patients aged ≥ 18 years at the initiation of VEN treatment were eligible for inclusion. While patients who had previously received treatment for AML were excluded, those who had undergone prior treatment with AZA for MDS were eligible for inclusion. Medical chart reviews were performed at the 10 sites, with a cut-off date of December 31, 2023. The demographics, clinical characteristics, treatment information, pathology findings, and outcome data for the patients were retrospectively collected by chart review, and then the data were de-identified and entered into electronic case report forms. The VENUS (UMIN ID: UMIN000050247) study was conducted in accordance with the principles of Good Epidemiology Practice, the applicable regulations and guidelines, the Declaration of Helsinki, and the “Ethical Guidelines for Medical and Health Research Involving Human Subjects” of the Ministry of Health, Labour, and Welfare, Japan. Before study initiation, ethics approval was obtained from the Japanese Red Cross Aichi Medical Center Nagoya Daiichi Hospital, Nagoya, Japan (approval number 2022–425). Given the retrospective design of the study and the difficulty in obtaining written consent from all patients, an opt-out system was adopted.

### Treatment

The patients were administered VEN on a ramp-up basis at the discretion of the treating physician, and the starting doses of VEN and AZA were also determined by the treating physician based on their package inserts. The doses were adjusted according to the potential for drug interactions, most commonly with anti-fungal agent. Specifically, VEN 200 mg was administered in combination with a moderately potent CYP3A4 inhibitors, such as fluconazole, and VEN 100 mg or 50 mg was administered with strong CYP3A4 inhibitors, the former with such as voriconazole and the latter with posaconazole, based on the package inserts. The final VEN dose and duration of administration were defined as the dose the patient was receiving and the duration of treatment at their last follow-up appointment. Cycle duration is defined as days from each cycle’s start to the next. For the final cycle, duration is calculated up to the earliest of discontinuation without VEN re-administration or final observation.

### Disease assessments

OS was calculated as the interval between a date of VEN administration and death, and it was censored on the date the patient was last known to be alive; survival was not censored at allogeneic stem cell transplantation (allo-HSCT). Event-free survival (EFS) was calculated as the interval between a date of VEN administration and relapse defined as progressive disease, treatment failure (failure to archive CR or < 5% bone marrow blasts in 6 months), confirmed relapse, or death. The response rate was calculated as the proportion of patients who achieved CR or CR with incomplete blood count recovery (CRi) at any time during the study, according to the modified International Working Group criteria for AML. The time to the first response was defined as the number of days from a confirmed diagnosis of AML to the earlier of CR or CRi. Cytogenetic and some molecular testing were performed at local or regional laboratories, as deemed appropriate by the treating physician. Wilms tumor 1 (*WT1*) mRNA testing was performed in peripheral blood (PB) using the *WT1* mRNA assay kit II (Otsuka Pharmaceutical Co., Ltd., Tokyo Japan) [24].

Blood cell counts were performed for patients meeting the following criteria: achievement of a blast percentage < 5% in the bone marrow, administration of more than one cycle of VEN + AZA, and availability of hematologic data for more than one cycle. The timing of blood cell counts was categorized as follows during each cycle: Day 1 (baseline), Days 8–14, Days 15–21, Days 22–28, and Days 29–35, and a mean count was calculated for each patient. A median value was then calculated for all the eligible patients.

### Statistical analysis

Continuous datasets are described using the median and range or mean. OS and EFS were estimated using the Kaplan–Meier method, and associations with prognostic factors were identified using the log-rank test. Cox proportional hazard regression analysis was then performed for OS. Missing data was not imputed. All analyses were conducted on data from all the eligible patients. The analyses were performed using SAS version 9.4 (SAS Institute, Cary, NC, USA).

## Results

### Patient characteristics

Data for a total of 120 consecutive adult patients who underwent VEN + AZA for newly diagnosed AML between June 2021 and September 2022 were analyzed. The median duration of follow-up was 13.6 months. The median age of the patients was 77 years (range, 31–93 years), and 66.7% were male (Table 1). A total of 58 patients (48.3%) had *de novo* AML, 62 (51.7%) had secondary AML (sAML), which was secondary to an antecedent hematologic disorder. Of those with sAML, 46 (74.2%) had antecedent MDS and 18 (39.1%) had a history of treatment of MDS with AZA. A total of 55 patients (45.8%) had a complex karyotype. Risk stratification according to the 2017 European LeukemiaNet (ELN) criteria was evaluated by physicians for 93 patient (77.5%) based on available data, including gene mutation (Supplemental Table S1a). Among these, 7 patients (5.8%) were classified as favorable risk, 41 patients (34.2%) as intermediate risks, and 45 patients (37.5%) as adverse risks. The most common WHO (2017) disease classification was AML-MRC (60.0%). We placed the patients with AML-MRC into one of two subgroups: “prior MDS,” defined as sAML with a history of MDS, and “*de novo* AML with AML-MRC,” consisting of those with *de novo* AML and myelodysplastic changes and/or MDS-related cytogenetic abnormalities (8.7% of − 5 or del(5q); − 7; − 17/abn(17p), 34.8% of complex karyotype and/or monosomal karyotype) (Supplemental Table S1a,b). Most of the patients (85.0%) were decided to be ineligible for standard chemotherapy because of their age (Supplemental Table S2).

**Table 1:** Patient characteristics

	Overall (*n* = 120)
Median age at diagnosis (years [range])	77(31–93)
Age categories—n (%)	
18– < 65	14 (11.7%)
65– < 75	31 (25.8%)
≥ 75	75 (62.5%)
Gender—*n* (%)	
Male	80 (66.7%)
Female	40 (33.3%)
AML type—*n* (%)	
De-novo	58 (48.3%)
Secondary	62 (51.7%)
MDS	46 (74.2%)
Past medication history of AZA	
Yes	18 (39.1%)
No	28 (60.8%)
MPN	5 (8.1%)
CMML	3 (4.8%)
Therapy related	8 (12.9%)
FAB classification	
M4 or M5	21 (17.5%)
Others	55 (45.8%)
Unknown	44 (36.7%)
AML classification-WHO 2017—*n* (%)	
AML with recurrent genetic abnormalities	3 (2.5%)
AML with myelodysplasia-related changes	72 (60.0%)
Therapy-related myeloid neoplasms	9 (7.5%)
AML, NOS	24 (20.0%)
Unknown	12 (10.0%)
Complex karyotype (*n*[%])	
Yes	55 (45.8%)
No	59 (49.2%)
Missing	6 (5.0%)
Baseline blood counts	
ANC (/µL), median	558
Hb (g/dL), median	8
Platelet (/µL), median	43,500
ELN risk stratification 2017*—*n* (%)	
Favorable	7 (5.8%)
Intermediate	41 (34.2%)
Adverse	45 (37.5%)
Unknown	27 (22.5%)
Allogenic transplant	13 (10.8%)

*AML* acute myeloid leukemia, *ANC* absolute neutrophil count, *AZA* azacitidine, *CMML* chronic myelomonocytic leukemia, *ELN* European LeukemiaNet, *FAB* French-American-British classification, *Hb* hemoglobin, *MDS* myelodysplastic syndrome, *MPN* myeloproliferative neoplasm, *NOS* not otherwise specified

^*^The availability of gene mutation data was limited per institution, and the ELN 2017 risk classification was assessed based on available data per physician assessment

### Therapy administered and supportive care required

#### Treatment schedule

The median duration between Day 1 of a cycle and Day 1 of the following cycle (median cycle length) was 38.5 days (range, 2–124 days) for cycle 1, 35 days (range, 7–105 days) for cycles 2, 35 days for cycles 6 (range, 9–173 days), and 35 days (range, 2–84 days) for cycle 10 (Table 2, Supplemental Fig. S1a). The median duration of VEN exposure in cycle 1 was 27 days (range, 2–52 days), with a median holding period of 12 days (range, 0–114 days). For cycle 2, the median VEN exposure was 22 days (range, 0–42 days), and for cycle 6, it was 21 days (range, 7–68 days). A similar pattern was observed in the *de novo* AML-MRC group, whereas the prior MDS group had medians of 26 days (range, 3–52 days) for cycle 1, 21 days (range, 0–35 days) for cycle 2, and a reduced exposure of VEN of 14.5 days (range, 8–28 days) for cycle 6 (Supplemental Fig. S1b,c, Supplemental Table S3).

**Table 2:** Treatment duration and dosing schedule

	Cycle 1 (*n* = 120)	Cycle 2 (*n* = 104)	Cycle 6 (*n* = 55)	Cycle 10 (*n* = 37)
Median days between day1 and next cycle (days)	38.5 (2,124)	35.0 (7,105)	35.0 (9,173)	35.0 (2,84)
VEN exposure duration, median (days)	27.0 (2,52)	22.0 (0,42)	21.0 (7,68)	14.0 (2,29)
VEN holding duration, median (days)	11.5 (0,114)	13.0 (0,89)	15.0 (0,159)	20.0 (0,77)
VEN dosage, mean (mg (SD))	280.6 (137.5)	236.3 (145.1)	235.5 (151.7)	208.1 (139.7)
VEN dose				
400 mg	64 (53.3%)	40 (39.2%)	23 (41.8%)	11 (29.7%)
below 400 mg	56 (46.7%)	62 (60.7%)	32 (58.2%)	26 (70.3%)
DDI	47 (83.9%)	58 (93.5%)	30 (93.8%)	20 (76.9%)
without DDI	9 (16.1%)	4 (6.5%)	2 (6.2%)	6 (23.1%)
AZA dose				
Dose/day, mean (mg/m^2^(SD))	73.75 (3.52)	73.53 (4.64)	69.77 (11.57)	69.55 (13.53)
Dosing days				
7 days	98 (81.7%)	81 (77.9%)	30 (55.6%)	17 (45.9%)
< 7 days	19 (15.8%)	19 (18.3%)	21 (39.0%)	19 (51.3%)
≥ 8 days	3 (2.5%)	4 (3.8%)	3 (5.6%)	1 (2.7%)

*AZA* azacitidine, *DDI* drug–drug interaction, *SD* standard deviation, *VEN* venetoclax

#### Dosage of VEN

The mean dose of VEN administered was 280.6 mg for cycle 1, and this gradually decreased from cycle 2 onward (Table 2). Of the patients, 53.3% were administered the standard dose of VEN (400 mg) during cycle 1 and 46.7% subsequently had their VEN dose reduced. Of these patients, 83.9% had their doses reduced because of drug–drug interactions (DDIs) involving drugs such as azoles, and 16.1% for other reasons, during cycle 1. With respect to DDIs, concomitant anti-fungal prophylaxis was performed in 35.8% with micafungin and in 47.4% with fluconazole during VEN + AZA treatment (Supplemental Table S4). During cycle 2, the proportions of the patients who were administered the standard dose of VEN (400 mg) were 39.2%, 52.8%, and 25.0% for the overall patient cohort, for the prior MDS, and *de novo* AML-MRC groups, respectively (Table 2, Supplemental Table S3).

#### Supportive treatment with G-CSF

The use of G-CSF for the patients who achieved remission, defined as < 5% blasts in bone marrow, was analyzed. The proportions of patients who received G-CSF post-remission were 98.5% (67/68), 80.8% (21/26), and 100% (16/16) for the overall patients who treated VEN + AZA, for the prior MDS, and *de novo* AML-MRC groups, respectively (Supplemental Table S5).

#### Recovery of blood cell counts

We analyzed the median neutrophil count according to treatment cycle for the patients who achieved < 5% blasts in bone marrow, and we found that this tended to recover from cycles 2–3 onward [19]. The duration of Grade 4 neutropenia (absolute neutrophil count [ANC] < 500 cells/μL) was > 21 days during cycle 1, and this decreased to ≤ 10 days from cycle 2 onward (Supplemental Fig.S2) in the overall patient cohort, and also in the prior MDS and *de novo* AML-MRC groups (Supplemental Fig.S3). The lowest median neutrophil count and the hemoglobin concentration tended to recover from cycle 2 onward in the overall patient cohort and in the prior MDS and *de novo* AML-MRC groups. The lowest median platelet counts remained relatively low in prior MDS, but a gradual recovery was identified in the overall patient cohort and in the *de novo* AML-MRC group (Supplemental Fig.S4).

### *Achievement of remission following VEN* + *AZA treatment*

**Fig. 1:**
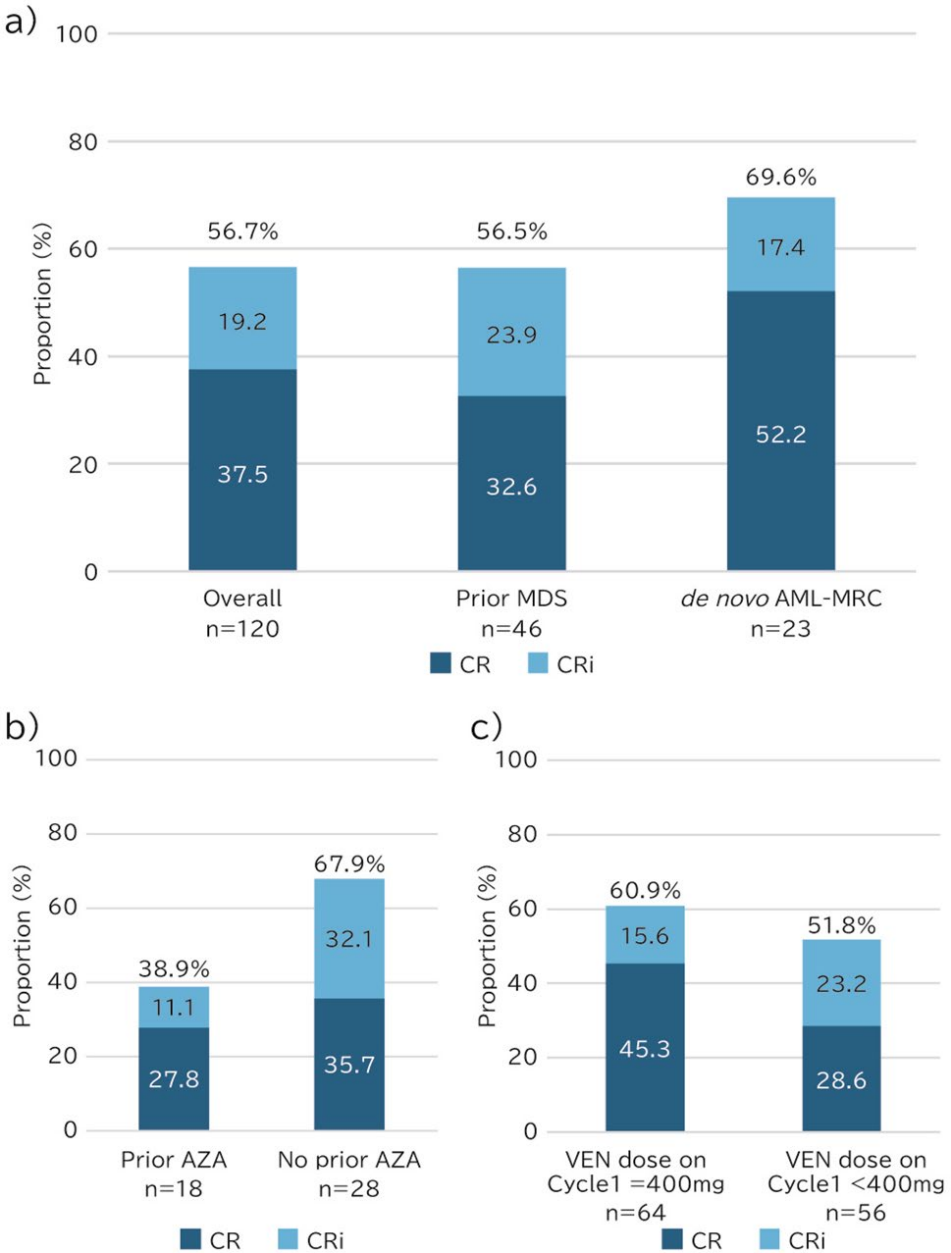
Clinical response rates. **a** Overall patients, prior MDS, and *de novo* AML-MRC groups. **b** In the prior MDS group, with or without prior AZA treatment. **c** VEN dose during cycle 1. *AML-MRC* acute myeloid leukemia with myelodysplasia-related changes, *AZA* azacitidine, *CR* complete remission, *CRi* complete remission with incomplete blood count recovery, *MDS* myelodysplastic syndrome, *VEN* venetoclax

**Fig. 2:**
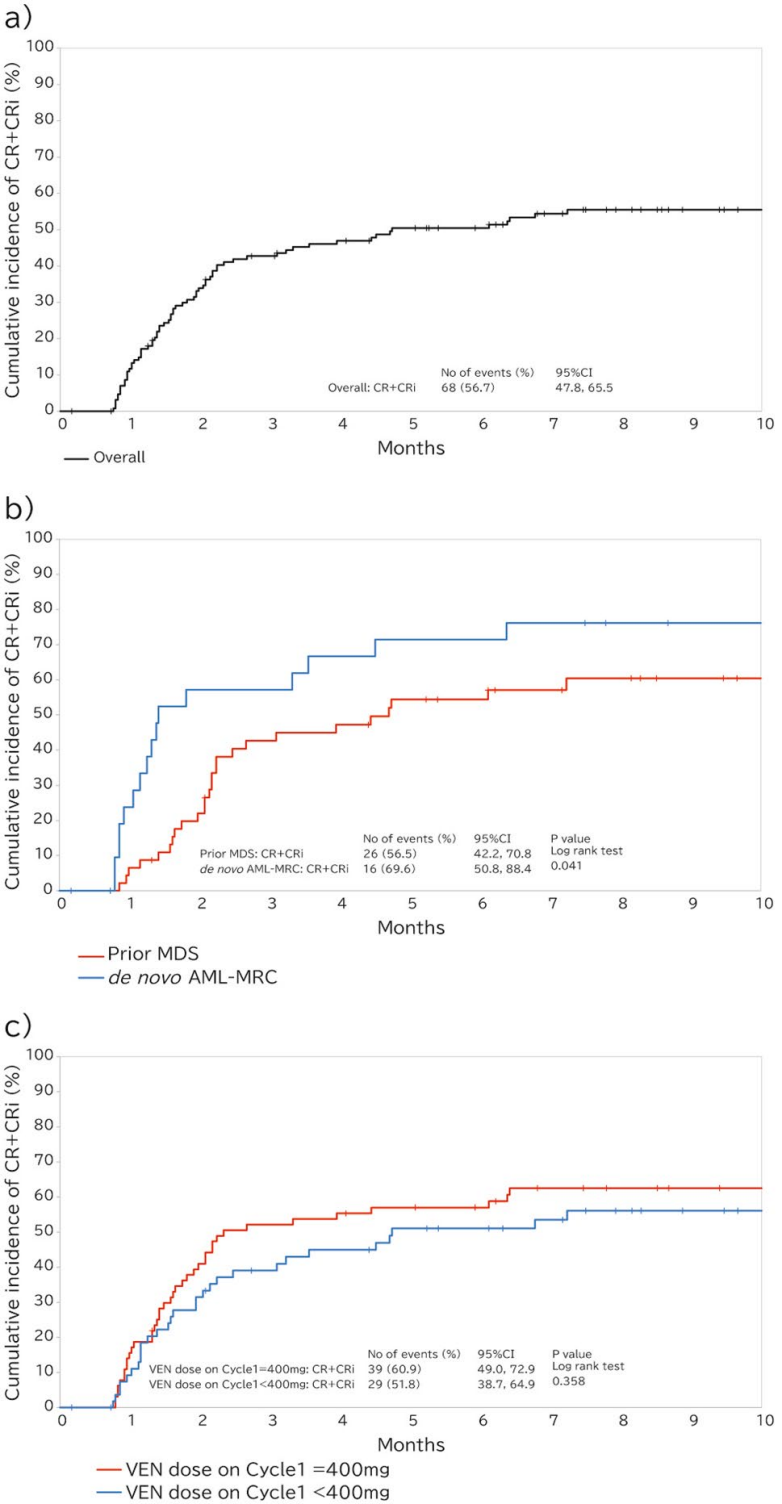
Cumulative incidence of the overall response. **a** Overall patients. **b** Prior MDS and *de novo* AML-MRC groups. **c** Patients who were administered the standard dose of VEN (400 mg) or ≤ 400 mg VEN during cycle 1. *AML-MRC* acute myeloid leukemia with myelodysplasia-related changes, *CI* confidence interval, *CR* + *CRi* complete remission with incomplete blood count recovery, *MDS* myelodysplastic syndrome, *VEN* venetoclax

Overall, the median duration of VEN treatment was 5.4 months (range, 0.1–29.3 months) and the median number of cycles was 5 (range, 1–28) (Table 3). A total of 37.5% achieved CR, and a further 19.2% achieved CRi, such that CR + CRi was achieved by 56.7% of the overall VEN + AZA cohort (Figs. 1a and 2, Supplemental Table S6). The CR + CRi rates in patients with prior MDS and *de novo* AML-MRC were 56.5% and 69.6%, respectively. The median times to CR + CRi were 1.6, 2.2, and 1.3 months for the overall patient cohort (Supplemental Table S6), those in the prior MDS, and *de novo* AML-MRC groups, respectively. The achievement of CR + CRi tended to be delayed in the prior MDS group, compared with that in the *de novo* AML-MRC group (Fig. 2b). In patients who had previously been treated with AZA for MDS, the prevalence of CR + CRi was 38.9% (Fig. 1b) and tended to be delayed achieving CR + CRi (Supplemental Fig.S5), whereas it was 67.9% in those who had not been treated. In patients who were administered the standard dose of VEN (400 mg) during cycle 1, the CR + CRi rate was 60.9% and the CR rate was 45.3%, whereas the CR + CRi rate was 51.8% and the CR rate was 28.6% for the patients who were administered < 400 mg VEN (Figs. 1c and 2c).

**Table 3:** Duration of treatment

	Overall (*n* = 120)	prior MDS (*n* = 46)	*De novo* AML-MRC (*n* = 23)
Duration of VEN treatment, months, median (range)	5.44 (0.07,29.31)	4.76 (0.20,20.40)	5.62 (0.07,29.31)
Number of treatment cycles, median (range)	5 (1,28)	4 (1,16)	5 (1,28)

*AML* acute myeloid leukemia, *AML-MRC* AML with myelodysplasia-related changes, *MDS* myelodysplastic syndrome, *VEN* venetoclax

### *Survival outcome associated with VEN* + *AZA therapy*

**Fig. 3:**
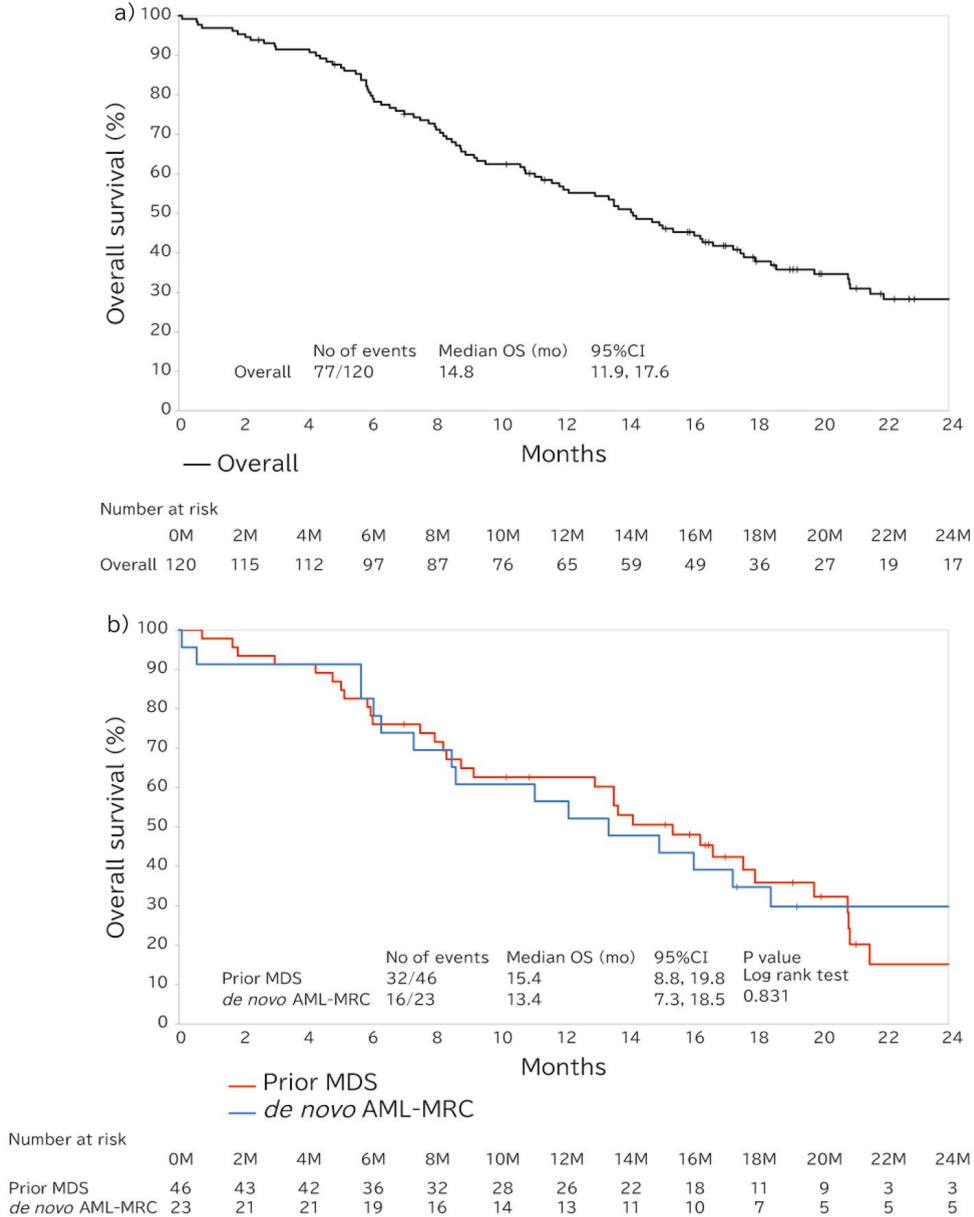
Overall survival. **a** Overall survival of the entire cohort. **b** Overall survival of the prior MDS and *de novo* AML-MRC groups. *AML-MRC* acute myeloid leukemia with myelodysplasia-related changes, *CI* confidence interval, *MDS* myelodysplastic syndrome, *OS* overall survival

**Fig. 4:**
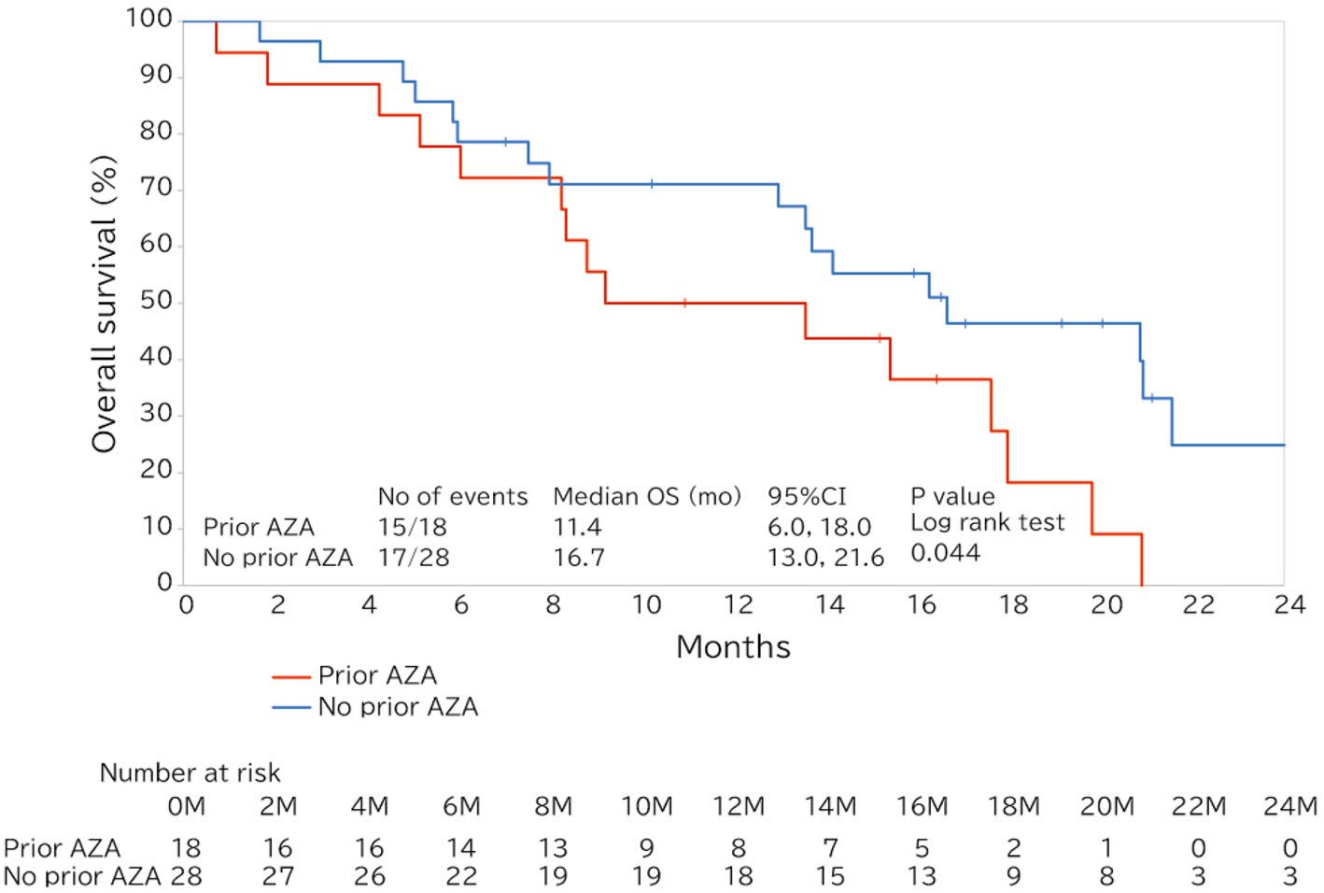
Overall survival of patients with prior MDS who had undergone prior treatment with AZA or not. *AML* acute myeloid leukemia, *AZA* azacitidine, *CI* confidence interval, *MDS* myelodysplastic syndrome, *OS* overall survival

The median OS for the overall patients who were treated VEN + AZA was 14.8 months (95% confidence interval [CI], 11.9–17.6 months) (Fig. 3a). For patients with the prior MDS and *de novo* AML-MRC, the median OS was 15.4 months (95% CI 8.8–19.8) and 13.4 months (95% CI 7.3–18.5), respectively, with no significant difference observed between the group (log-rank, *p* = 0.831) (Fig. 3b). In addition, the median EFS of the two groups was comparable (prior MDS: 13.6 months [95% CI 7.1–17.6]; *de novo* AML-MRC: 13.4 months [95% CI 5.7–18.5 months]), *p* = 0.607) (Supplemental Fig.S6). The median OS of the patients who administered concomitant G-CSF post-remission was 17.3 months (95% CI 14.2–20.9), 16.7 months (95% CI 13.6–20.9), and 16.7 months (95% CI 11.1–not estimable [NE]) in the overall patients who treated VEN + AZA and those in the prior MDS and *de novo* AML-MRC groups, respectively (Supplemental Fig.S7). For the patients who had previously been treated with AZA for MDS demonstrated shorter median OS of 11.4 months (95% CI 6.0–18.0 months) compared to 16.7 months (95% CI 13.0–21.6 months) for those who had not been treated (log-rank, *p* = 0.044) (Fig. 4).

We also evaluated the relationship of *WT1* mRNA expression in PB with OS. Several previous studies have shown that high PB *WT1* mRNA expression after the treatment of AML was associated with relapses and a poor prognosis [24–27]. The median OS of the patients who achieved *WT1* mRNA negativity, indicated by lower than 50 copies/μg RNA in PB, was 18.5 months (95% CI 13.4–NE), whereas it was 14.8 months (95% CI 10.6–20.9) for those who did not (log-rank, *p* = 0.271) (Supplemental Fig. S8a). *WT1* mRNA negativity was achieved by 2 (6.7%) patients by the end of cycle 1, 21 (70.0%) patients by the end of cycle 4, 5 (16.7%) patients by the end of cycle 7, and 2 (6.7%) patients after cycle 7 (Supplemental Fig. S8b).

Univariable Cox regression showed that a complex karyotype was associated with poor survival (Table 4). Thirteen patients underwent subsequent allo-HSCT following VEN + AZA treatment, and landmark analysis of OS from the time of allo-HSCT was conducted for these patients and median OS was not reached (Supplemental Fig. S9).

**Table 4:** Univariate Cox regression analysis

Univariate Cox regression analysis				
		Univariate		
Variable		HR (95%CI)		
Age	65–74	REF		
	75–84	0.996	[0.567–1.747]	0.988
	≥ 85	1.782	[0.891–3.563]	0.102
Complex karyotype	NO	REF		
	YES	2.561	[1.602–4.095]	< 0.001
VEN dose in cycle 1	< 400 mg/d	REF		
	400 mg/d	0.729	[0.466–1.142]	0.168
Neutrophil count	≥ 500	REF		
	< 500	1.102	[0.704–1.727]	0.671
Platelet count	≥ 25,000	REF		
	< 25,000	1.637	[1.004–2.669]	0.048
Hb	≥ 8.0	REF		
	< 8.0	1.364	[0.869–2.140]	0.177

*CI* confidence interval, *Hb* hemoglobin, *HR* hazard ratio, *REF* reference, *VEN* venetoclax

## Discussion

Here, we have reported the outcomes of VEN + AZA treatment in patients with newly diagnosed AML in Japan. Despite differences in the demographics of the included patients from those in the VIALE-A randomized trial, in particular, a higher incidence of sAML and a higher prevalence of a history of MDS and treatment with AZA, comparable results were obtained with respect to survival. In practice, real-world outcomes often do not match those obtained in clinical trials, likely because of the complexities in managing VEN. Real-world evidence from early versus later post-approval periods suggests an impact of improved treatment management following established protocols on patient outcomes. Although the present cohort included patients who had been treated with VEN shortly after its marketing (VEN was approved in March 2021 in Japan, and we included patients who started VEN treatment between June 2021 and September 2022), similar clinical outcomes to those of the VIALE-A study were obtained. This suggests that VEN doses in accordance with protocol or labeling recommendations were administered at recommended intervals. In addition, we have provided detailed information regarding the doses and timing of VEN and AZA administration, as well as supportive care and blood cell count recovery, which may inform treatment management in addition to established protocols/label recommendation for clinicians who are administering these drugs.

In our cohort, patients with a history of MDS had a lower response rate. This might be because patients with a history of AZA treatment had a lower response rate. These comprised approximately 40% of the prior MDS group, and had a CR + CRi prevalence of 38.9%, compared with 67.9% for those with no history of AZA treatment. Thus, previous AZA administration may reduce the responsiveness of patients to VEN + AZA [22, 23]. Otherwise, patients with MDS with history of AZA treatment may have inherently higher risks of disease and aggressive disease than those without such a history. Therefore, the interpretation of the efficacy of treatment for those with or without a history of AZA treatment should be approached carefully.

Furthermore, patients with prior MDS required a longer period of time to achieve CR + CRi than those with *de novo* AML-MRC. However, although patients with prior MDS took longer for their first response, this response was more rapid than that to AZA monotherapy among IC-ineligible patients with AML [4]. In addition, no differences were identified between the two groups in terms of OS or EFS. This may be speculated that in patients with prior MDS, the response to treatment may be slower or poorer than in those with *de novo* AML-MRC, but the continued administration of VEN + AZA may be effective in the long term. Moreover, the median OS of the patients with prior MDS has been demonstrated to be comparable to that reported in previous studies [22, 28].

Administering the standard dose of VEN of 400 mg from cycle 1 tended to be associated with a higher response rate. However, among the patients receiving less than 400 mg, some had their doses modified because of azole drug interactions to an equivalent of 400 mg, and others who had their doses reduced for other reasons. The former group received appropriately adjusted doses on the basis of recommendation for concomitant use of CYP3A4 inhibitor, unlike the latter group. In addition, intrinsic variability in the concentrations of azoles, such as voriconazole or posaconazole, may occasionally require therapeutic drug monitoring [29] and suggests that VEN concentration may be influenced by drug interactions or reduced its clearance [30].

Post-remission G-CSF use could support to maintain treatment cycles with shorter delays of treatment and it has been recommended for cytopenia management during VEN + AZA treatment [4, 5, 18, 31]. In the present cohort, although many of the patients were treated immediately after the launch of VEN in Japan, those who achieved remission were administered G-CSF. The proportion of the post-remission G-CSF use was high across the entire cohort of patients who had been treated with VEN + AZA, as well as among those with prior MDS and newly developed AML-MRC, and this did not have a negative effect on the OS of patients with such disease backgrounds.

We found that the duration of grade 4 neutropenia was reduced to < 10 days from cycle 2 onward, following remission, and there were no differences in this trend between patients with prior MDS and those with *de novo* AML-MRC. Thus, prolonged neutropenia of > 10 days’ duration [32], which is associated with a high risk of invasive fungal infection, is less likely to occur, regardless of disease background, if appropriate measures, such as a maintenance of VEN dose and optimal G-CSF use, are implemented following remission. However, in this analysis, the number of evaluable patients decreased with each subsequent cycle, and it should be considered a limitation that patients for relapse or serious adverse events may have discontinued the treatment over time. In addition, the median nadir ANC and post-remission hemoglobin concentrations of patients who had previously been administered AZA and those with newly developed AML-MRC exhibited similar trends. However, a trend toward a lower platelet count in patients with prior MDS has been demonstrated previously, and this may be explained by the characteristics of the MDS or previous AZA therapy [33].

The importance and utility of measurable residual disease (MRD) monitoring as a prognostic factor for OS in VEN + AZA have been highlighted previously [34]. Multicolor flow cytometry and RT-PCR methods are recommended to assess MRD [35]. However, these techniques are only available at specific facilities in Japan. Alternatively, *WT1* mRNA, a biomarker that is expressed at high levels in AML, is used to assess MRD in real-world clinical practice in Japan [26, 27].

The described *WT1* expression thresholds have not been standardized, which poses a challenge for its objective evaluation. A 1-log reduction in *WT1* has previously been described as an indicator of a response to VEN treatment [25], and in the present study, *WT1* monitoring was only feasible in 41 patients (data not shown). However, those who achieved a 1-log reduction by cycle 3 tended to exhibit superior OS. Furthermore, this 1-log reduction in *WT1* was attained relatively quickly, with approximately 80% of patients achieving this by cycle 4, reflecting the rapid response to VEN. However, according to Pratz et al., approximately 80% of responders achieve MRD negativity (< 10^−3^) by cycle 7, suggesting that treatment intensity should be maintained until this time to obtain a good response [34].

In the present study, approximately 10% of the patients underwent allo-HSCT following their treatment (data not shown). Landmark OS analysis demonstrated favorable outcomes with the median OS of the patients who underwent allo-HSCT was not reached. These results suggest that VEN + AZA treatment could be a potential therapeutic option bridging allo-HSCT, and may contribute to the long-term survival of patients, including those with AML who are ineligible for IC but eligible for transplantation [36].

The present study had several limitations. First, because it was a retrospective analysis, we cannot exclude the possibility that other confounding variables, particularly relating to prior AZA exposure, may have influenced the results. Second, the power of some of the comparisons was limited by small sample sizes. A number of patients had not undergone molecular testing, which is limited in clinical practice in Japan, and therefore did not undergo accurate ELN assessment. Lastly, data could not be collected regarding infections, and particularly invasive fungal infections, in the context of the administration of anti-fungal prophylaxis.

In conclusion, we have presented real-world evidence regarding the clinical outcomes of the VEN + AZA treatment of IC-ineligible patients with AML in Japan. In patients with a history of AZA treatment, who were excluded from the VIALE-A trial, we identified an OS that was comparable to that of clinical trials. This suggests that the VEN doses and schedule administered were in accordance with protocol or labeling recommendations, and that cytopenia in these patients were effectively managed. No differences in effectiveness were identified between the patients with prior MDS and those with *de novo* AML-MRC. In addition, when managed according to protocol or label recommendations, the severity of neutropenia did not differ and remained manageable. However, further detailed analyses of the efficacy of VEN + AZA and patient management according to their genetic abnormalities associated with MDS are warranted.

## Supplementary Information

Below is the link to the electronic supplementary material.

Supplementary file1 (PDF 1412 KB)

## Data Availability

The datasets used and/or analyzed during the current study are available from the corresponding author on reasonable request.
