# Supplementary material for: Real-world outcomes of venetoclax and azacitidine in Japanese patients with newly diagnosed acute myeloid leukemia (VENUS study)
Source: Int J Hematol. 2025 Nov 7;123(2):178–88. doi: 10.1007/s12185-025-04093-y (PMC12913348; doi:10.1007/s12185-025-04093-y)
Supplement: Supplementary file 1 — Supplementary file1 (PDF 1412 KB) [file 12185_2025_4093_MOESM1_ESM.pdf]

Supplementary material

Table S1a. Cytogenetic and genetic mutation risk according to ELN 2017

	Overall (n=120)	Prior MDS (n=46)	*de novo* AML-MRC (n=23)
Favorable			
t(8;21)(q22;q22.1); RUNX1-RUNX1T1	1 (0.8%)	0 (0%)	0 (0%)
inv(16)(p13.1q22) or t(16;16)(p13.1;q22); CBFB-MYH11	1 (0.8%)	0 (0%)	0 (0%)
Mutated NPM1 without FLT3-ITD or with FLT3-ITD low	3 (2.5%)	1 (2.2%)	1 (4.3%)
Biallelic mutated CEBPA	2 (1.7%)	1 (2.2%)	0 (0%)
Intermediate			
Mutated NPM1 and FLT3-ITD high	0 (0%)	0 (0%)	0 (0%)
Wild-type NPM1 without FLT3-ITD or with FLT3-ITD low without adverse-risk genetic lesions	7 (5.8%)	1 (2.2%)	3 (13.0%)
t(9;11)(p21.3;q23.3); MLLT3-KMT2A	0 (0%)	0 (0%)	0 (0%)
Cytogenetic abnormalities not classified as favorable or adverse	36 (30.0%)	12 (26.1%)	5 (21.7%)
Adverse			
t(6;9)(p23;q34.1); DEK-NUP214	0 (0%)	0 (0%)	0 (0%)
t(v;11q23.3); KMT2A rearranged	0 (0%)	0 (0%)	0 (0%)
t(9;22)(q34.1;q11.2); BCR-ABL1	0 (0%)	0 (0%)	0 (0%)
inv(3)(q21q26.2) or t(3;3)(q21;q26.2); GATA2, MECOM(EVI1)	0 (0%)	0 (0%)	0 (0%)
-5 or del(5q); -7; -17/abn(17p)	5 (4.2%)	1 (2.2%)	2 (8.7%)
Complex karyotype, monosomal karyotype	35 (29.2%)	16 (34.8%)	8 (34.8%)
Wild-type NPM1 and FLT3-ITDhigh	4 (3.3%)	3 (6.5%)	0 (0%)
Mutated RUNX1	2 (1.7%)	1 (2.2%)	1 (4.3%)
Mutated ASXL1	4 (3.3%)	1 (2.2%)	1 (4.3%)
Mutated TP53	3 (2.5%)	1 (2.2%)	2 (8.7%)
Unknown	27 (22.5%)	11 (23.9%)	4 (17.4%)

Table S1b. AML-MRC group, defined as secondary or *de novo* AML (n,%)

	Overall (n=120)
AML with myelodysplasia-related changes	72 (60.0%)
Secondary	49 (68.1%)
MDS-Overt	43 (59.7%)
Treatment-related	1 (1.4%)
CMML-overt	3 (4.2%)
MPN-overt	2 (2.8%)
de novo AML-MRC	23 (31.9%)

AML, acute myeloid leukemia; AML-MRC, AML with myelodysplasia-related changes; CMML, chronic myelomonocytic leukemia; ELN, European LeukemiaNet; MDS, myelodysplastic syndrome; MPN, myeloproliferative neoplasm

Table S2. Reasons for ineligibility for standard chemotherapy (n,%)

Age	102 (85.0%)
Concurrent disease	24 (20.0%)
Other	15 (12.5%)

Table S3. Duration of treatment and dosing schedule for patients in the prior MDS (A) and *de novo* AML-MRC (B) groups

(A) Patients in the prior MDS group

	Cycle 1 (n=46)	Cycle 2 (n=38)	Cycle 6 (n=18)	Cycle 10 (n=12)
Median days between day1 and next cycle (days)	36.5 (3,119)	34.0 (7,91)	35.0 (14,56)	35.0 (10,56)
VEN dosing duration, median (days)	26.0 (3,52)	21.0 (0,35)	14.5 (8,28)	14.0 (8,29)
VEN holding duration, median (days)	8.0 (0,109)	12.5 (0,70)	15.5 (0,42)	17.5 (0,39)
VEN dosage, mean (mg (SD))	267.4 (144.2)	261.1 (156.8)	252.8 (159.5)	220.8 (145.3)
VEN dose				
400mg	23 (50.0%)	19 (52.8%)	9 (50.0%)	4 (33.3%)
below 400mg	23 (50.0%)	17 (47.2%)	9 (50.0%)	8 (66.7%)
DDI	21 (91.3%)	16 (94.1%)	8 (88.9%)	6 (75.0%)
without DDI	2 (8.7%)	1 (5.9%)	1 (11.1%)	2 (25.0%)
AZA dose				
Dose/day, mean (mg/m^2(SD))	73.22, (4.09)	73.00, (4.25)	66.17, (15.18)	70.08, (12.03)
Dosing days				
7 days	36 (78.3%)	24 (63.2%)	9 (41.2%)	4 (50.0%)
<7 days	9 (19.5%)	12 (31.5%)	9 (41.2%)	4 (50.0%)
≥8 days	1 (2.2%)	2 (5.3%)	1 (5.9%)	0 (0%)

(B) Patients in the *de novo* AML-MRC group

	Cycle 1 (n=23)	Cycle 2 (n=20)	Cycle 6 (n=11)	Cycle 10 (n=8)
Median days between day1 and next cycle (days)	40.0 (2,55)	34.5 (15,84)	32.0 (28,173)	41.5 (28,84)
VEN dosing duration, median (days)	27.0 (2,46)	22.0 (10,42)	21.0 (8,68)	14.0 (7,21)
VEN holding duration, median (days)	9.0 (0,33)	12.0 (0,73)	10.0 (0,159)	27.0 (10,77)
VEN dosage, mean (mg (SD))	287.0 (128.1)	215.0 (124.7)	218.2 (157.0)	218.8 (162.4)
VEN dose				
400mg	12 (52.2%)	5 (25.0%)	4 (36.4%)	3 (37.5%)
below 400mg	11 (47.8%)	15 (75.0%)	7 (36.6%)	5 (62.5%)
DDI	7 (63.6%)	14 (93.3%)	6 (85.7%)	4 (80.0%)
without DDI	4 (36.3%)	1 (6.7%)	1 (14.3%)	1 (20.0%)
AZA dose				
Dose/day, mean (mg/m^2(SD))	73.65, (3.79)	73.48, (3.98)	71.91, (7.75)	68.63, (15.75)
Dosing days				
7 days	19 (82.6%)	18 (90.0%)	6 (54.5%)	4 (50.0%)
<7 days	3 (13.0%)	1 (5.0%)	3 (27.3%)	4 (50.0%)
≥8 days	1 (4.3%)	1 (5.0%)	2 (18.2%)	0 (0%)

AML-MRC, acute myeloid leukemia with myelodysplasia-related changes; AZA, azacitidine; DDI, drug-drug interaction; MDS, myelodysplastic syndrome; SD, standard deviation; VEN, venetoclax

Table S4. Concomitant use of antifungal prophylaxis

	Overall (n=120)		Cycle 1	
	n	%	n	%
Any concomitant use of antifungal	95	79.2	88	73.3
Fluconazole	45	47.4	39	44.3
Voriconazole	25	26.3	19	21.6
Itraconazole	16	16.8	13	14.8
Posaconazole	12	12.6	8	9.1
Micafungin	34	35.8	29	33.0
Caspofungin	2	2.1	2	2.3
Amphoterisin B	8	8.4	4	4.5

Table S5. Concomitant use of G-CSF in patients who achieved CR

	Overall	Prior MDS	*de novo* AML-MRC
Patient who achieved <5% blasts in BM	n=68	n=26	n=16
G-CSF received, n (%)	67 (98.5%)	21 (80.8%)	16 (100%)

AML-MRC, acute myeloid leukemia with myelodysplasia-related changes; BM, bone marrow; CR, complete remission; G-CSF, granulocyte colony-stimulating factor; MDS, myelodysplastic syndrome

Table S6. Rate of CR+CRi and the time to achieve a response

	Overall			Prior MDS			*de novo* AML-MRC		
	N=120			N=46			N=23		
CR+CRi Rate (as best response) - n (%) [95% CI]									
CR	45	(37.5)	[28.8, 46.2]	15	(32.6)	[19.1, 46.2]	12	(52.2)	[31.8, 72.6]
CRi	23	(19.2)	[12.1, 26.2]	11	(23.9)	[11.6, 36.2]	4	(17.4)	[1.9, 32.9]
CR+CRi	68	(56.7)	[47.8, 65.5]	26	(56.5)	[42.2, 70.8]	16	(69.6)	[50.8, 88.4]
Subjects with Best Response of CR+CRi - Median [range]									
Time to First Response (months)									
CR+CRi	1.6 [0.8-7.2]			2.2 [0.9-7.2]			1.3 [0.8-6.4]		

AML-MRC, acute myeloid leukemia with myelodysplasia-related changes; CI, confidence interval; CR, complete remission; CRi, complete remission with incomplete blood count recovery; MDS, myelodysplastic syndrome

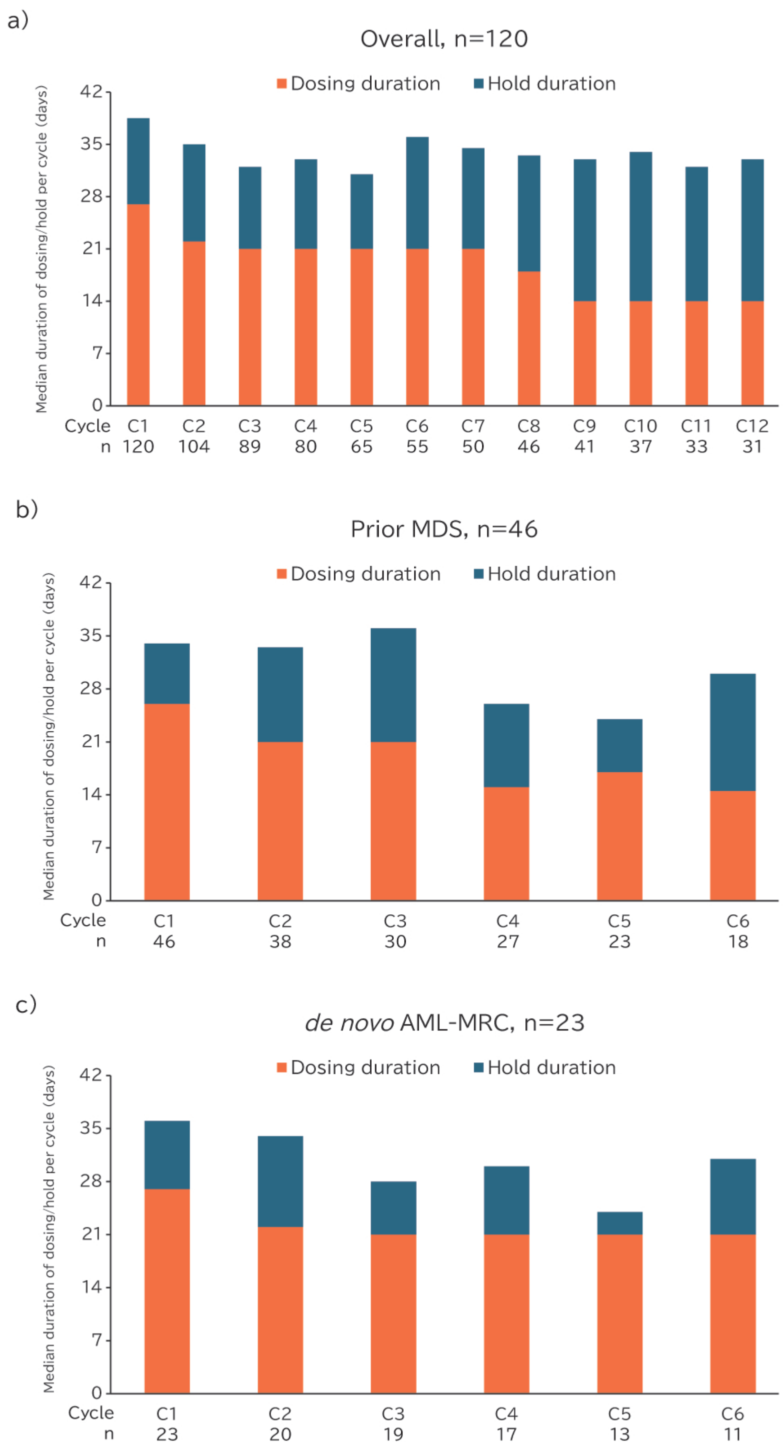

Figure S1. VEN dosing schedule

a) Overall patients. b) Prior MDS group. c) *De novo* AML-MRC group.

AML-MRC, acute myeloid leukemia with myelodysplasia-related changes; MDS, myelodysplastic syndrome; VEN, venetoclax

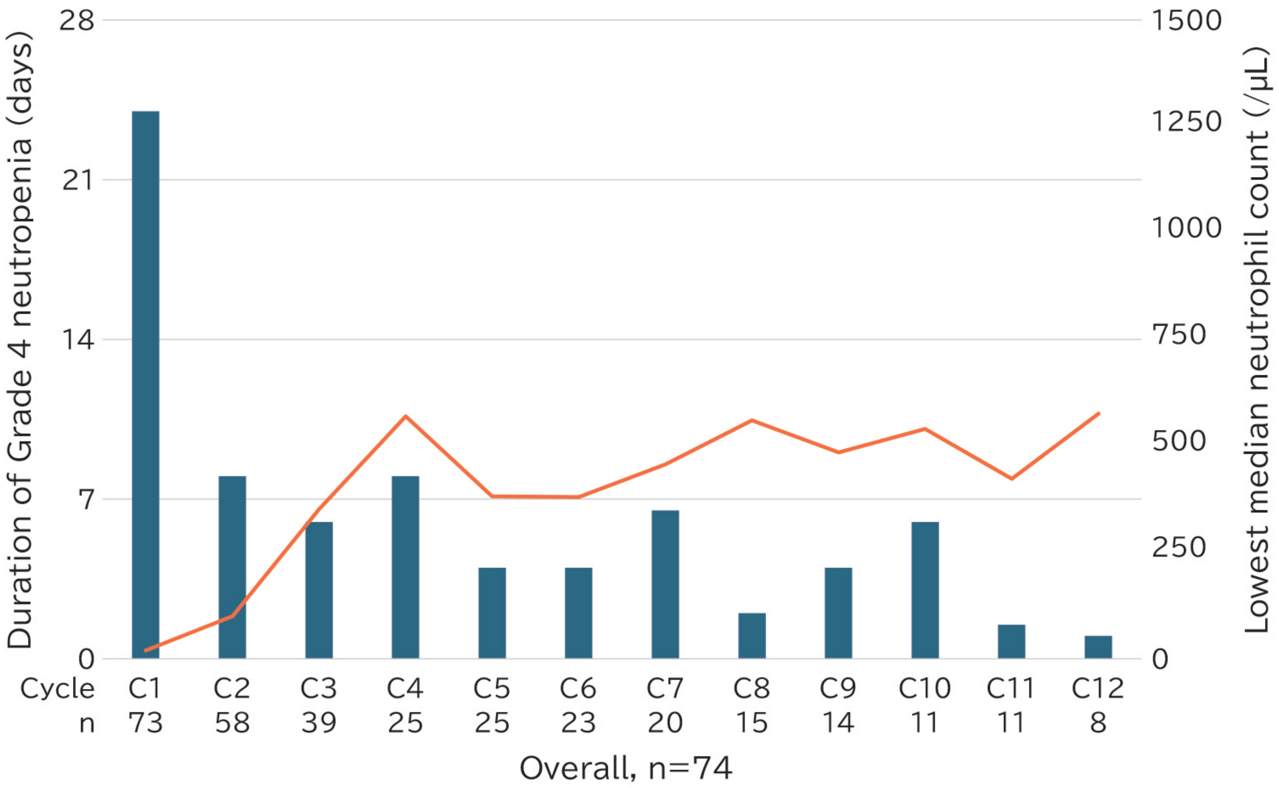

Figure S2. Duration of Grade 4 (absolute neutrophil count < 500 cells /µL) neutropenia and the median lowest neutrophil count in patients who achieved <5% blasts in bone marrow

Bar graph indicated the duration of Grade 4 neutropenia per cycle and the line graph indicated the median lowest neutrophil counts.

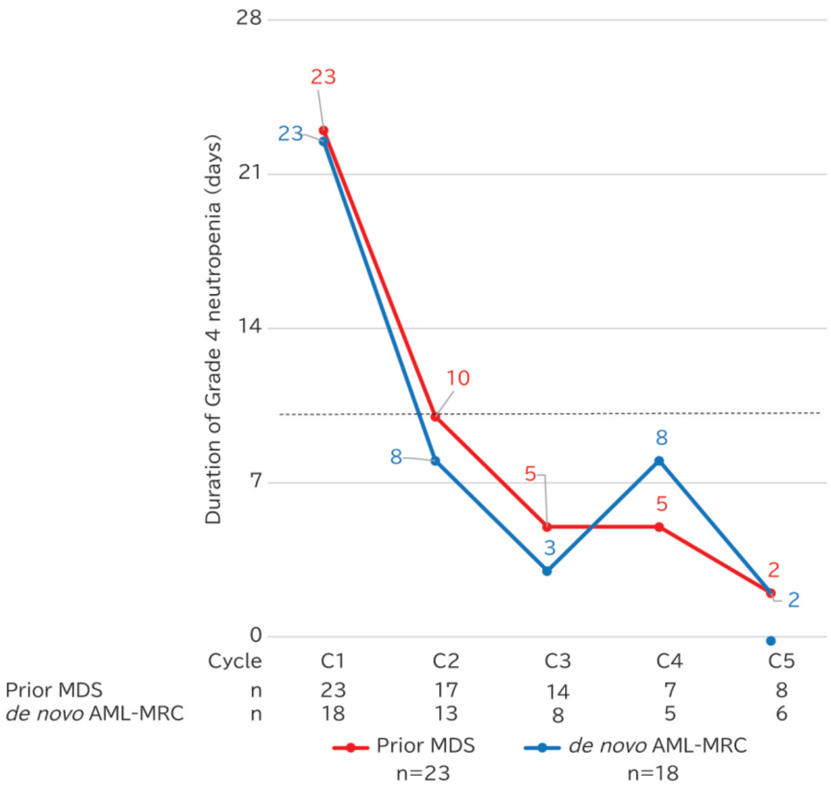

Figure S3. Duration of Grade 4 neutropenia who achieved <5% blasts in bone marrow

AML-MRC, acute myeloid leukemia with myelodysplasia-related changes; MDS, myelodysplastic syndrome

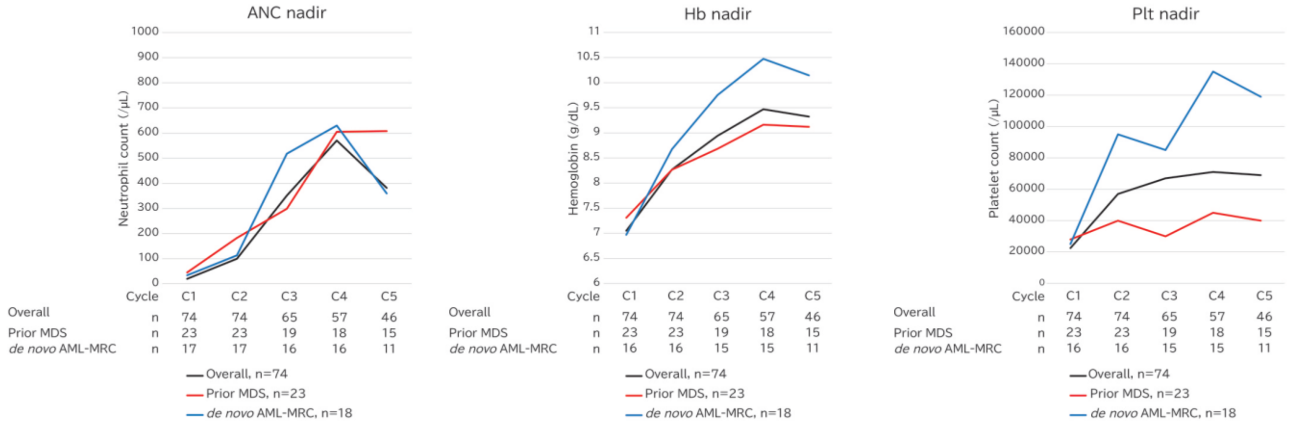

Figure S4. Lowest median blood counts during each cycle in patients who achieved blasts <5% in their bone marrow

AML-MRC, acute myeloid leukemia with myelodysplasia-related changes; ANC, absolute neutrophil count; Hb, hemoglobin; MDS, myelodysplastic syndrome; Plt, platelet

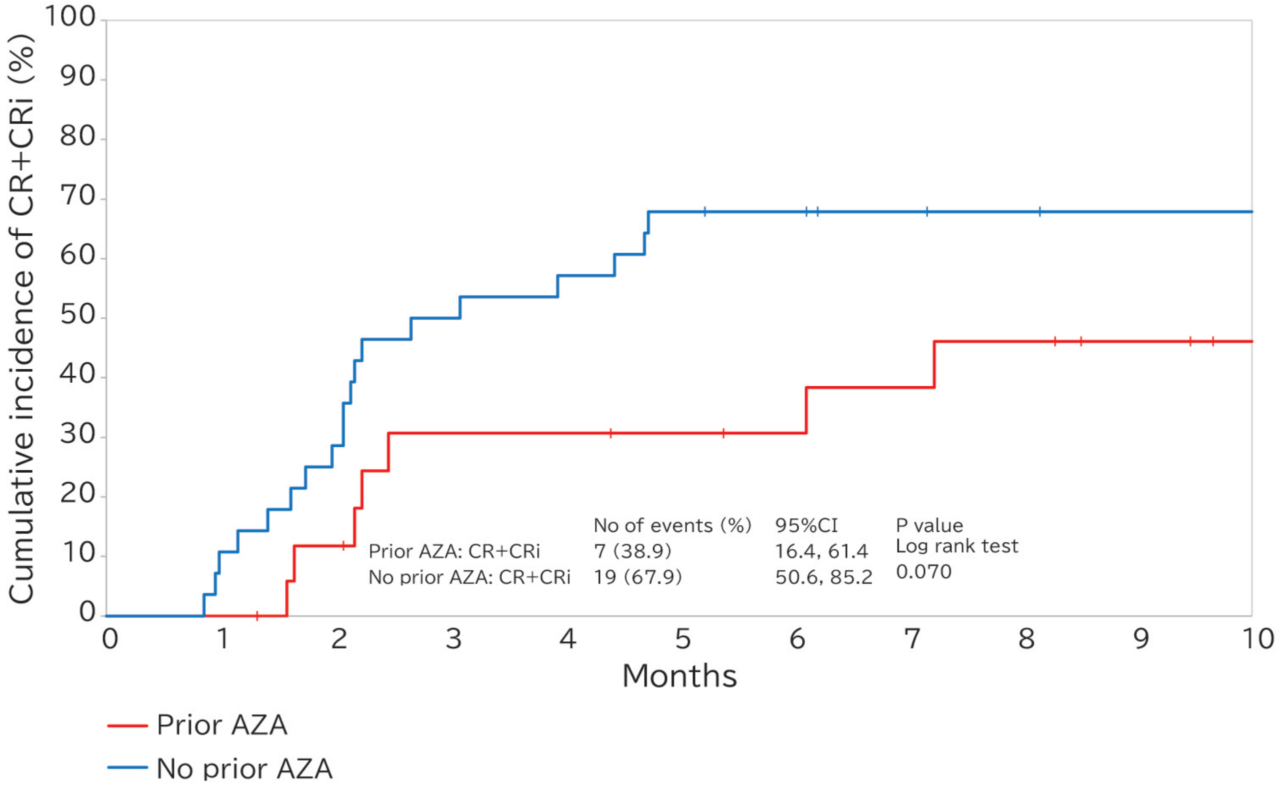

Figure S5. Cumulative incidence of the CR+CRi in the prior MDS group, according to prior AZA treatment status

AZA, azacitidine; CI, confidence interval; CR+CRi, complete remission with incomplete blood count recovery; MDS, myelodysplastic syndrome

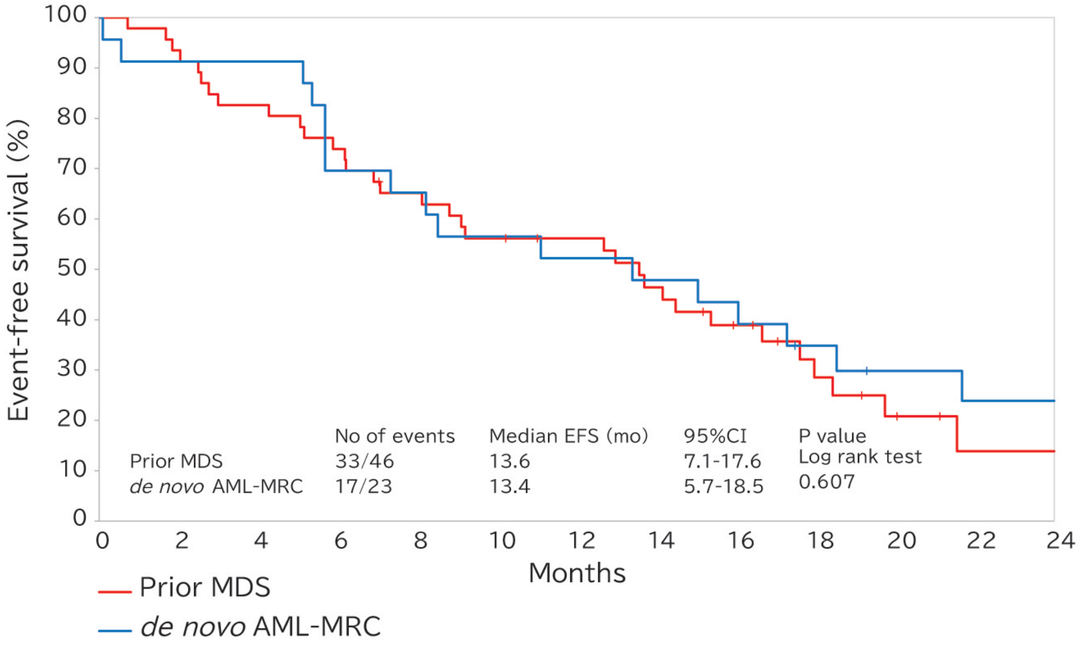

Number at risk

	0M	2M	4M	6M	8M	10M	12M	14M	16M	18M	20M	22M	24M
Prior MDS	46	43	38	34	29	25	23	19	14	8	5	2	2
de novo AML-MRC	23	21	21	16	15	13	12	11	10	7	5	4	4

Figure S6. Event-free survival of the patients in the prior MDS and *de novo* AML-MRC groups
AML-MRC, acute myeloid leukemia with myelodysplasia-related changes; CI, confidence interval; EFS, event-free survival; MDS, myelodysplastic syndrome

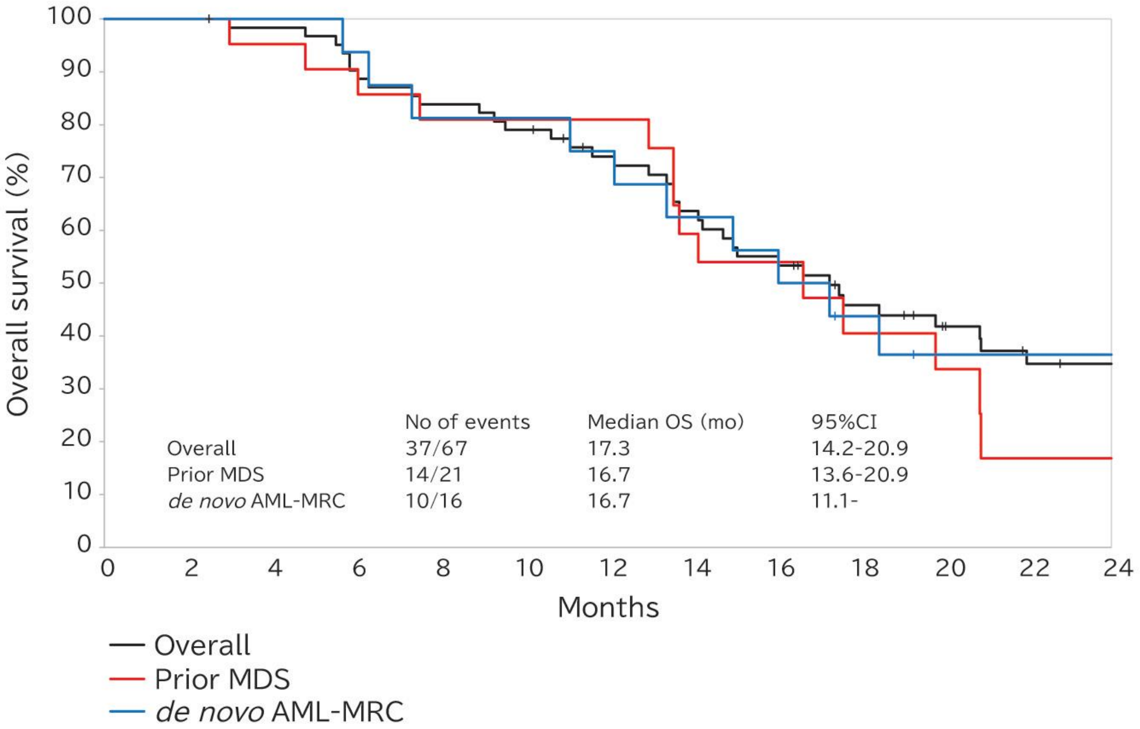

Number at risk

	0M	2M	4M	6M	8M	10M	12M	14M	16M	18M	20M	22M	24M
Overall	67	67	61	56	52	49	43	37	32	24	19	14	12
Prior MDS	21	21	20	19	17	17	15	11	10	6	5	2	2
de novo AML-MRC	16	16	16	15	13	13	12	10	9	6	4	4	4

Figure S7. Overall survival of participants who were concomitantly administered G-CSF and achieved CR

AML-MRC, acute myeloid leukemia with myelodysplasia-related changes; CI, confidence interval; CR, complete remission; EFS, event-free survival; G-CSF, granulocyte colony-stimulating factor; MDS, myelodysplastic syndrome

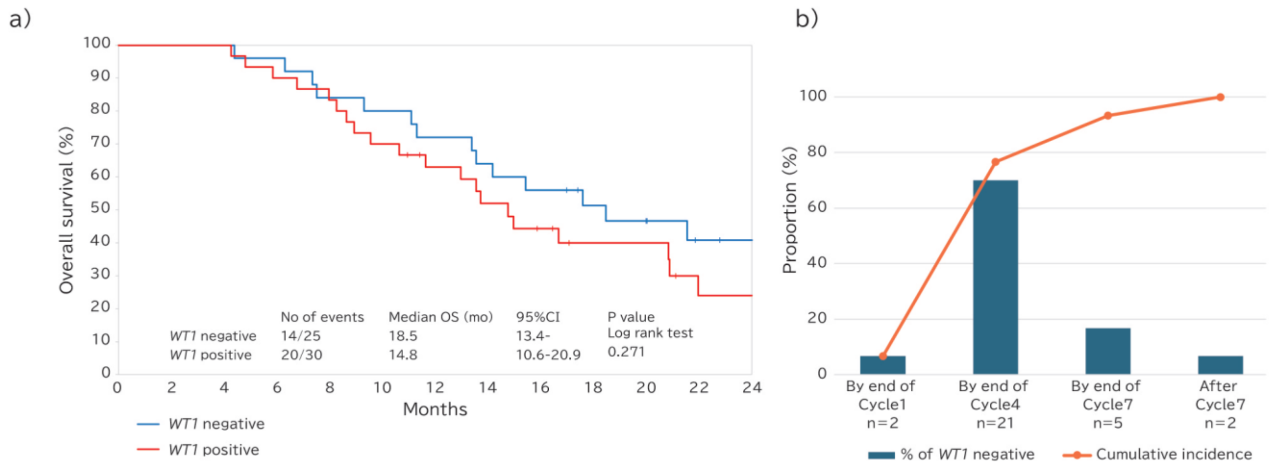

Number at risk

	0M	2M	4M	6M	8M	10M	12M	14M	16M	18M	20M	22M	24M
WT1 negative	25	25	25	24	21	20	18	16	14	11	9	6	5
WT1 positive	30	30	30	27	25	21	17	14	11	8	8	4	4

Figure S8. Overall Survivals in WT1 mRNA reduction. a) Overall survival of patients based on WT1 mRNA negativity (< 50 copies/ug RNA) achievement. b) Cumulative incidence of a WT1 negativity, according to the treatment cycle

CI, confidence interval; OS, overall survival; WT1, Wilms tumor 1

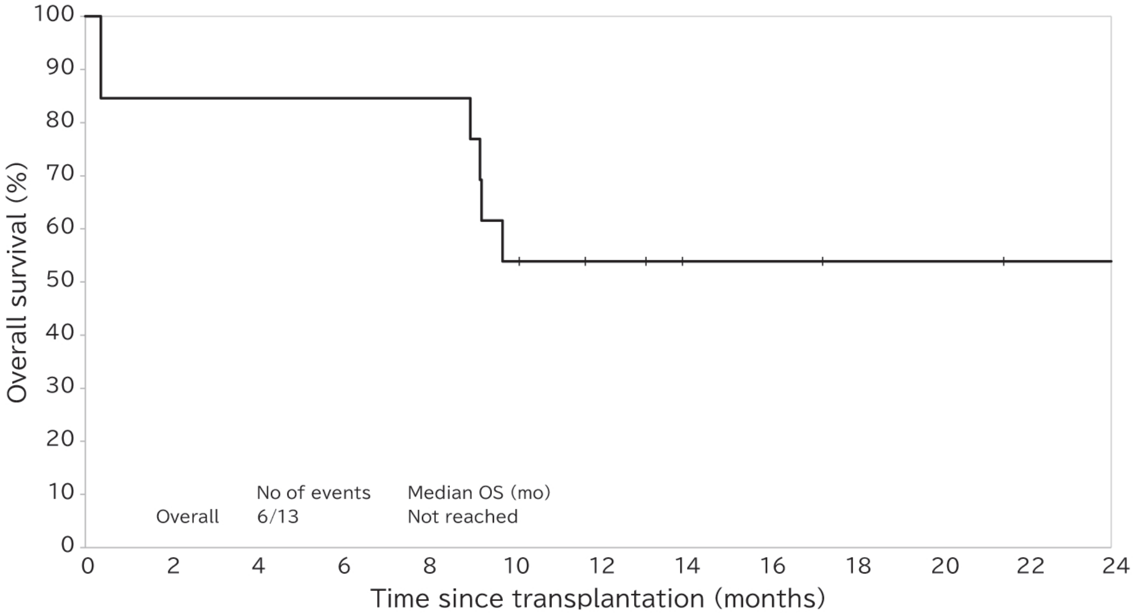

Number at risk

	0M	2M	4M	6M	8M	10M	12M	14M	16M	18M	20M	22M	24M
Overall	13	11	11	11	11	7	5	3	3	2	2	1	1

Figure S9. Landmark survival analysis from the time of allo-HSCT.

Allo-HSCT, allogenic hematopoietic stem cell transplantation; OS, overall survival
